# Running Stride Length And Rate Are Changed And Mechanical Efficiency Is Preserved After Cycling In Middle-Level Triathletes

**DOI:** 10.1038/s41598-019-54912-6

**Published:** 2019-12-05

**Authors:** Rodrigo Gomes da Rosa, Henrique Bianchi de Oliveira, Luca Paolo Ardigò, Natalia Andrea Gomeñuka, Gabriela Fischer, Leonardo Alexandre Peyré-Tartaruga

**Affiliations:** 1 Exercise Research Laboratory, Universidade Federal do Rio Grande do Sul, Porto Alegre, Brazil; 2 Department of Neurosciences, Biomedicine and Movement Sciences, School of Exercise and Sport Science, University of Verona, Verona, Italy; 3 Departamento de Investigación de la Facultad de Ciencias de la Salud, Universidad Católica de las Misiones (UCAMI), Posadas, Argentina; 4 Laboratory of Biomechanics, Departamento de Educação Física, Universidade Federal de Santa Catarina, Florianópolis, Brazil

**Keywords:** Physiology, Metabolism

## Abstract

Although cycling impairs the subsequent metabolic cost and performance of running in some triathletes, the consequences on mechanical efficiency (*Eff*) and kinetic and potential energy fluctuations of the body center of mass are still unknown. The aim of this study was to investigate the effects of previous cycling on the cost-of-transport, *Eff*, mechanical energy fluctuations (W_tot_), spring stiffness (K_leg_ and K_vert_) and spatiotemporal parameters. Fourteen middle-level triathletes (mean ± SD: maximal oxygen uptake, $\dot{{\rm{V}}}$O_2max_ = 65.3 ± 2.7 ml.kg^−1^.min^−1^, age = 30 ± 5 years, practice time = 6.8 ± 3.0 years) performed four tests. Two maximal oxygen uptake tests on a cycle ergometer and treadmill, and two submaximal 20-minute running tests (14 km.h^−1^) with (prior-cycling) and without (control) a previous submaximal 30-minute cycling test. No differences were observed between the control and post-cycling groups in *Eff* or W_tot_. The *Eff* remains unchanged between conditions. On the other hand, the K_vert_ (20.2 vs 24.4 kN.m^−1^) and K_leg_ (7.1 vs 8.2 kN.m^−1^, p < 0.05) were lower and the cost-of-transport was higher (p = 0.018, 3.71 vs 3.31 J.kg^−1^.m^−1^) when running was preceded by cycling. Significantly higher stride frequency (p < 0.05, 1.46 vs 1.43 Hz) and lower stride length (p < 0.05, 2.60 vs 2.65 m) were observed in the running after cycling condition in comparison with control condition. Mechanical adjustments were needed to maintain the *Eff*, even resulting in an impaired metabolic cost after cycling performed at moderate intensity. These findings are compatible with the concept that specific adjustments in spatiotemporal parameters preserve the *Eff* when running is preceded by cycling in middle-level triathletes, though the cost-of-transport increased.

## Introduction

Mechanical efficiency (*Eff*) is defined as the ratio of total mechanical energy output to the total metabolic energy input and, therefore, integrates physiologic and biomechanical features^1–3^. Furthermore, the total mechanical work (W_tot_) represents the mechanical energy fluctuations from the body and segment center of mass, performed primarily by the muscles and tendons during running^4^. Competitive distance runners may produce similar levels of external work with lower net energy expenditure and, thus, run at a higher *Eff* ^5^. Nonetheless, the effects of previous cycling on running *Eff* have not been verified in triathletes. The determination of W_tot_ and *Eff*, besides metabolic economy may be useful to understand the role of running technique on running performance.

Running cost-of-transport (CoT) is defined as the energy amount to cover a given distance commonly expressed in J.kg^−1^.m^−1^. It has been documented that CoT is one of the determinants of long-distance running performance^6–8^. The CoT of triathletes is affected by cycling in a level-dependent manner. In elite triathletes, the CoT decreased by 3.7% and increased by 2.3% in middle-level triathletes^9^. Two mechanisms were associated with to these findings: *(i*) a better metabolic economy of the respiratory muscles and, *(ii)* better stiffness regulation observed among the elite triathletes but not among their less successful counterparts^4,5^.

According to the spring-mass model system, the combination of passive (tendons and intramuscular connective tissues) and active (muscles) structures are responsible for elastic energy recovery during running^10^. The arch of the foot stores enough energy to make running more efficient and the metabolic energy saved by the arch is largely explained by the passive-elastic work it supplies that would otherwise be done by active muscle^11–13^, and, recently it was found that elastic bouncing was optimized in runners of the best performance level^14^. Although it is difficult to specify the extent to which the elastic mechanism affects the CoT and *Eff*, higher W_tot_ and *Eff* are related to improvements in parameters of spring-mass model. It is possible to measure the parameters of this system through vertical stiffness (K_vert_*)* and leg stiffness (K_leg_), which allow an estimate of how compliant the system is with respect to, e.g., fatigue or prior exercise^15,16^. In middle and elite triathletes, no significant changes in K_leg_, K_vert_ stride length, and stride frequency were reported between the initial and final stages of an Olympic distance, specifically in triathletes who adopted the same running speed during these two stages^17^.

Hypothetically in a specific triathlon situation, the previous cycling could decrease the running W_tot_ worsening the running *Eff*. Also, the decrease in the spring-mass stiffness after cycling would offer indirect evidence of the impairment of the running elastic mechanism. Consequently, there would be an increase in the metabolic component due to the decline in elastic energy return. The purpose of this study was to test the hypothesis that the augmentation in the CoT is related to changes in mechanical parameters (W_tot_, K_leg_, and K_vert_) and decrease in *Eff*. The testing of these hypotheses may contribute towards a better design of training for triathletes.

## Methods

### Participants

Fourteen triathletes participated (average ± SD: age = 30 ± 5 years; body mass = 74.2 ± 6.8 kg; stature = 179.1 ± 7.3 cm; $\dot{{\rm{V}}}$O_2max_ = 65.3 ± 2.7 ml.kg.^−1^min^−1^; triathlon practice time = 6.8 ± 3.0 years; weekly distance training (km) - cycling: 260 ± 40; running: 43 ± 9; swim: 11 ± 3). These triathletes had regional level, defined, therefore, as middle level triathletes. The choice for these athletes was based on local availability. The inclusion criteria were: *(i)* having at least 2 years of experience in the triathlon, *(ii)* training for at least 12 h per week in the last year; and *(iii)* age > 18 years. The exclusion criterion included any orthopedic or musculoskeletal injury. The sample size was calculated using Winpepi software (version 4.0), in which a significance level of 0.05 and a power of 90% were adopted using the CoT, K_leg_, and K_vert_ variables^8,9,17,18^. The probability is 90 percent that the study will detect a treatment difference at a two-sided 0.05 significance level, if the true difference between conditions is 0.19 J.kg^−1^.m^−1^ (smallest meaningful difference). All participants read and signed the informed consent form approved by the Ethics Committee of Federal University of Rio Grande do Sul (No. 579.277) before participating in the study. All experiments were performed in accordance with the Declaration of Helsinki.

**Figure 1:**
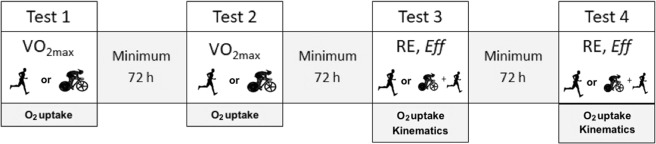
Experimental design. Tests were performed on four different days: In the first and second session, the $\dot{{\rm{V}}}$O_2max_ tests were performed in randomized order (running or cycling). After these sessions, the submaximal tests were carried out, also in randomized order (running or cycling + running). Each session was separated by an interval of at least 72 hours.

Subjects performed four tests: two incremental tests (running and cycling) and two submaximal constant-speed running tests, both in random order (Fig. 1).

### Maximal tests

The running and cycling maximal tests were performed to determine the maximal oxygen uptake ($\dot{{\rm{V}}}$O_2max_) and confirm the metabolic intensity of subsequent submaximal tests. The subjects were prepared for the test by placement of a heart rate monitor Polar S810 (Polar Electro Oy, Kempele, Finland) and neoprene mask for gas collection. The heart rate, $\dot{{\rm{V}}}$O_2_, carbon dioxide ($\dot{{\rm{V}}}$CO_2_), and ventilation per minute ($\dot{{\rm{V}}}$E) were continuously measured by indirect calorimetry throughout an automated gas analysis system (VO2000, Medgraphics, St Paul, Minnesota, USA) validated accordingly^19^. The auto-calibration method of the system was executed daily before every test. Calibration of the calorimeter was carried out according to the manufacturer instructions. Every 5 days of data collection, a known gas mixture (5.01 CO_2_, 16.02 O_2_, balance N_2_) was inserted into the system for simulation. In the running tests, subjects warmed up by walking on a treadmill for 6 minutes at 6 km.h^−1^, and the initial load was 10 km.h^−1^ with a 1% grade. The speed was increased by 1 km.h^−1^ every 3 minutes until volitional exhaustion. In the cycling maximal tests, the initial load was 150 W 6 minutes and the subjects’ bikes were positioned on a cycle ergometer (Computrainer, ProLab 3D, Racermate Inc., Seattle, USA). After the warm up, the power output was increased by 25 W.min^−1^ and cadence was maintained near 90 rpm and controlled by visual feedback until volitional exhaustion or until the subjects could not maintain the cadence of at least 70 rpm.

### Submaximal tests

The gases were calibrated before every test. The ventilatory data were registered during whole test using the same automated gas analysis system utilized in the maximal tests. The tests started with the collection of $\dot{{\rm{V}}}$O_2_ and $\dot{{\rm{V}}}$CO_2_ in a standing position for six minutes. The warm up followed the same protocol described in the maximal tests. In the ‘submaximal running after cycling’ test, the athletes’ bikes were fixed on a cycle ergometer and the athletes sustained a pedaling power of 10% below the ventilatory threshold for 30 minutes. The athletes were asked to make the transition from bike to run as fast as possible (<1 minute), and then perform the submaximal running test at 14 km.h^−1^ for 20 minutes. In all submaximal running tests the steady-state $\dot{{\rm{V}}}$O_2_ was reached.

Concurrently with the collection of ventilatory parameters, kinematic data were recorded using a motion capture system (Vicon Motion Systems, United Kingdom) operating at 100 Hz^20^. Six infrared Bonita and Tseries cameras registered the tridimensional positions of eighteen reflexive markers (14 mm diameter), fixed on the following anatomical landmarks: fifth metatarsal, calcaneus, lateral malleolus, femoral epicondyle, greater trochanter, acromion, lateral epicondyle of the humerus, middle point ulnar-radius, and temporal bone^21^. Kinematic data were collected in one-minute durations four times, at the 3^rd^−5^th^, 8^th^-10^th^, 13^th^-14^th^, 18^th^-20^th^ minute.

### Processing data

#### $\dot{V}$O_2max_ and ventilatory threshold

$\dot{{\rm{V}}}$O_2max_ was determined as a plateau in $\dot{{\rm{V}}}$O_2_ despite an increase in power output, a 1.15 respiratory exchange ratio, or an heart rate over 90% of the predicted maximal heart rate^22^. The ventilatory threshold^23^ was determined as follows: i) a regular rise in the ventilatory equivalent of $\dot{{\rm{V}}}$O_2_; ii) a concomitant nonlinear rise in the ventilatory equivalent of $\dot{{\rm{V}}}$CO_2_; and iii) a decrease in the difference in the inspired and end-tidal oxygen pressure. Two blinded experienced researchers located the ventilatory threshold by visual inspection.

#### Mechanical work (W_tot_), stiffness and spatiotemporal parameters

The body center of mass and location and body segment mass and locations were estimated^24^. We calculated the mechanical energy fluctuations considering the external (W_ext_) and internal (W_int_) counterparts as proposed by Cavagna and Kaneko^1^. The total external energy of the body center of mass is given as a function of time by the point-to-point sum of the gravitational potential and kinetic energies. The sum of positive increments from the total external energy curve results in the W_ext_. The mechanical energy fluctuations of the segments relative to the body center of mass were calculated. Additionally, the W_int_ was considered as the sum of the positive increments for each body segment separately, with an exception for arm plus upper arm and shank plus thigh, therefore, allowing intralimb energy transfer^25^. The W_tot_ is calculated as |W_ext_| + |W_int_|, and all mechanical energy variables are divided by body mass and stride length, hence, the unit is J.kg^−1^.m^−1^. The mechanical power (P_mec_) was also calculated by dividing the W_tot_ by speed (in m.s^−1^) and was expressed in W.kg^−1^.

The stride length and frequency were determined by algorithms built in Nexus software^26,27^. The maximal vertical force (F_max_) from calculated the basis modelling of F(t) curves by a simple sinus function, vertical oscillation during the step (ΔL), vertical oscillation during contact (Δyc), K_vert_ and K_leg_ were calculated according to estimations proposed by Morin and colleagues as follows^28^.

$${{\rm{F}}}_{\max ={\rm{mg}}\frac{{\rm{\pi }}}{2}(\frac{{\rm{Tf}}}{{\rm{Tc}}}+1)}$$

where m is body mass, and g is gravitational acceleration. The Δyc is determined as follows:

$$\Delta {y}_{c}=\frac{{{\rm{F}}}_{max}\cdot {{\rm{Tc}}}^{2}}{{\rm{m}}\,{{\rm{\pi }}}^{2}}+{\rm{g}}\frac{{{\rm{Tc}}}^{2}}{8}$$

Therefore, the Kvert is calculated as follows:

$${{\rm{K}}}_{{\rm{vert}}=}{{\rm{F}}}_{{\rm{\max }}}\cdot {{\Delta {\rm{y}}}_{{\rm{c}}}}^{-1}.$$

Also, the stiffness of the leg spring (K_leg_ in kN.m^−1^) was calculated as follows:

$${{\rm{K}}}_{{\rm{leg}}=}{{\rm{F}}}_{{\rm{\max }}}\cdot {\Delta {\rm{L}}}^{-1}$$

where ΔL is the maximum leg spring compression (in m) calculated from values of initial leg length L (great trochanter to ground distance in a standing position), running velocity (v, in m.s^−1^), contact time (Tc, in s), and vertical maximal downward displacement of the body center of mass during contact Δy.

$$\Delta {\rm{L}}={\rm{L}}-\sqrt{{{\rm{L}}}^{2}-(\frac{{\rm{v}}.{{\rm{Tc}}}^{2}}{2})+{\Delta {\rm{y}}}_{c}}.$$

#### Cost-of-transport (CoT) and mechanical efficiency (Eff)

The $\dot{{\rm{V}}}$O_2_ of exercise was averaged from the 60 seconds at each stage (3^rd^−5^th^, 8^th^−10^th^, 13^th^–14^th^, 18^th^–20^th^ minute) and was subtracted from $\dot{{\rm{V}}}$O_2_ at rest. The running economy was denoted by CoT, expressed in J.kg^−1^.m^−1^. We divided the net metabolic rate (gross – stand metabolic rate) by speed^29,30^, and we converted oxygen in ml to Joules relative to combustion enthalpy of substrates resulting from oxidation observed indirectly from the respiratory exchange ratio^31^. The metabolic power (Pmet) was also calculated and expressed in W.kg^−1^. *Eff* is defined as the fraction of the CoT that is transformed into W_tot_^1^, algebraically defined as *Eff* = W_tot_.CoT^−1^. All data can be seen in the electronic Supplementary Material (ESM 1).

### Statistical analysis

Descriptive statistics were analyzed with mean and standard deviation. The specific tests below were used to compare the dependent variables between running-after-cycling versus just-running conditions at four stages throughout the submaximal running tests. Shapiro-Wilk’s and Levene’s tests confirmed the normality and the homogeneity of the variances, respectively. A paired t-test was used to compare the means of the dependent variables with and without previous cycling, and a one-way ANOVA was used to compare the dependent variables at different stages of submaximal tests with a Bonferroni *post hoc* test to locate the differences. The significance level adopted was α = 0.05 and all data were processed in the SPSS version 17.0 statistical package.

## Results

**Figure 2:**
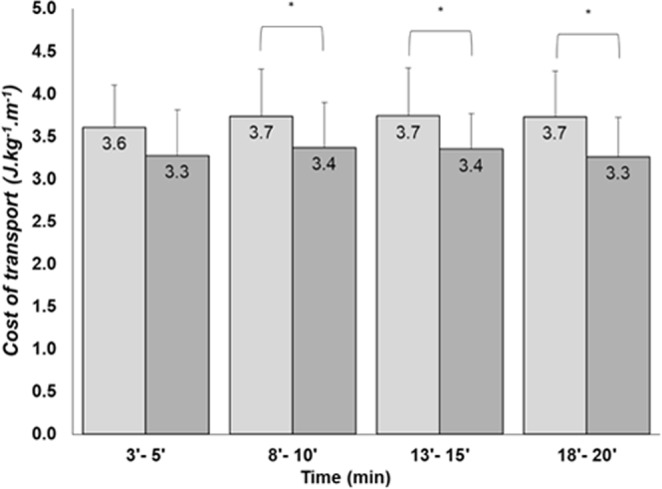
Mean and standard-deviation values of cost-of-transport during running at 14 km.h^−1^, the light gray and dark gray bars represent the cost of transport the post-cycling running and control, respectively. Asterisks denote values significantly different (p < 0.05) between conditions.

Figure 2 shows the CoT data. CoT was increased with previous cycling and was kept constant during the four stages of submaximal running tests. Therefore, the triathletes performed the submaximal tests running in a moderate metabolic domain (corresponding to approximately 72% $\dot{{\rm{V}}}$O_2max_ and 82% ventilatory threshold).

**Figure 3:**
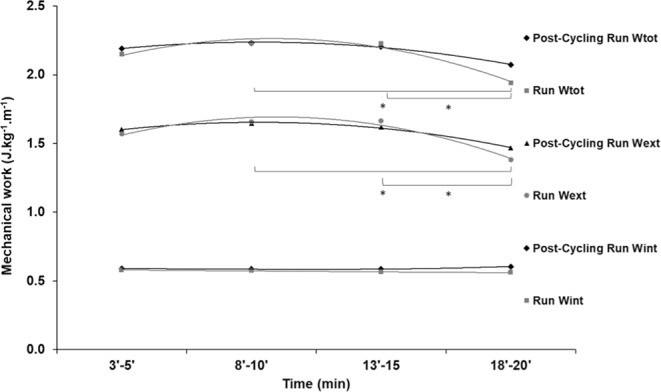
Mean values of mechanical energy fluctuations during the 20-min running at 14 km.h^−1^. The grey symbols represent the running without the previous cycling. The black symbols represent the running with previous cycling. The squares represent the values of internal mechanical work (W_int_). The diamonds represent the values of external mechanical work (W_ext_). The circles represent the values of total mechanical work (W_tot_). Standard deviations have been omitted for clarity. Asterisks denote values significantly different (p < 0.05) between conditions.

No significant differences were found in W_int_ (p > 0.05) between running with or without previous cycling, nor with the time factor (Fig. 3). The running W_int_ values on average were 0.59 J.kg^−1^.m^−1^ with previous cycling and 0.57 J.kg^−1^.m^−1^ without previous cycling. The W_ext_ values also remained unchanged between running with and without previous cycling (p > 0.05). However, W_ext_ and W_tot_ were lower at the final stage compared to those at the second and third stages (p < 0.05) only for running without previous cycling (p < 0.05). The P_met_ and P_mec_ values are listed in the electronic Supplementary Material (ESM 2).

**Figure 4:**
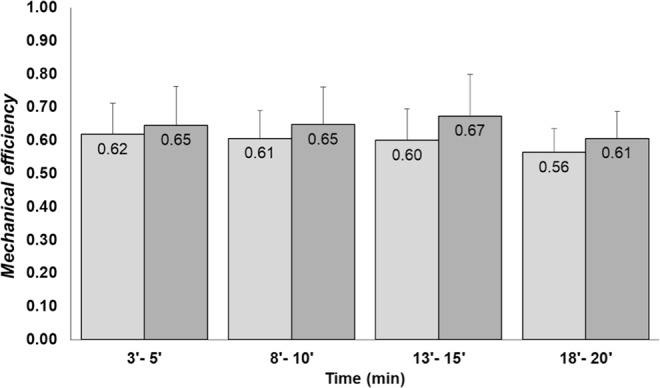
Mechanical efficiency (*Eff*) in the 20-minute submaximal constant-speed running test at 14 km.h^−1^. The light gray and dark gray bars represent the mean and standard deviation of *Eff* in Post-cycling run and control run, respectively. *Represents p < 0.05 of the paired t-test.

The *Eff* remained unchanged along the test independently of conditions. Also, the previous cycling was not capable of promoting changes in the *Eff* in comparison with control condition (p > 0.05, Fig. 4).

### Spring-mass and spatiotemporal parameters

The stride length and frequency, K_leg_ and K_vert_ did not change across all sections of the tests (p > 0.05; Table 1). However, the triathletes decreased their K_leg_ and K_vert_ at the end of the test between running after cycling and running without previous cycling. They increased their cadence and reduced their stride length systematically when they ran with previous cycling in comparison to the control condition. While the contact and flight times were similar between conditions in the first three sections, in the last section the contact time was higher and the flight time was lower in the previous cycling condition than those in the control condition. In addition, higher F_max_ and smaller ΔL and Δyc values were found in the last section in the previous cycling condition compared to the control condition.

**Table 1:** The mechanical parameters are presented as mean ± SD in the four sections of submaximal running tests following control and post-cycling run. *Significantly different from previous cycling condition, p < 0.05.

Time (min)	3′–5′		8′–10′		13′–14′		18′–20′	
	Run cycling	Run control	Run cycling	Run control	Run cycling	Run control	Run cycling	Run control
SL (m)	2.59 ± 0.10	*2.65 ± 0.12	2.59 ± 0.10	2.64 ± 0.11	2.60 ± 0.09	*2.65 ± 0.10	2.61 ± 0.11	*2.65 ± 0.12
SF (Hz)	1.46 ± 0.06	*1.43 ± 0.06	1.46 ± 0.06	*1.43 ± 0.06	1.45 ± 0.05	*1.43 ± 0.06	1.45 ± 0.06	*1.43 ± 0.06
T_c_ (s)	0.253 ± 0.026	0.255 ± 0.030	0.250 ± 0.022	0.254 ± 0.024	0.250 ± 0.020	0.247 ± 0.026	0.263 ± 0.024	*0.245 ± 0.021
T_f_ (s)	0.092 ± 0.017	0.093 ± 0.021	0.090 ± 0.015	0.097 ± 0.013	0.093 ± 0.012	0.099 ± 0.015	0.088 ± 0.018	*108 ± 0.018
K_leg_ (kN.m^−1^)	7.37 ± 1.42	7.31 ± 1.42	7.65 ± 1.60	7.25 ± 1.27	7.62 ± 1.23	7.98 ± 1.23	7.08 ± 1.39	*8.25 ± 1.49
K_vert_ (kN.m^−1^)	22.77 ± 3.27	21.78 ± 3.77	22.41 ± 3.59	21.96 ± 3.94	21.95 ± 2.87	22.09 ± 1.98	20.17 ± 1.96	*24.44 ± 2.89
F_max_ (N)	1597 ± 149	1585 ± 163	1612 ± 145	1609 ± 150	1602 ± 78	1650 ± 125	1565 ± 105	*1712 ± 154
Δy_c_ (m)	0.215 ± 0.035	0.223 ± 0.037	0.210 ± 0.029	0.213 ± 0.035	*0.215 ± 0.030	*0.210 ± 0.040	0.230 ± 0.036	*0.200 ± 0.026
Δ*L* (m)	0.080 ± 0.010	0.079 ± 0.013	0.081 ± 0.013	0.081 ± 0.012	0.083 ± 0.007	0.076 ± 0.008	0.089 ± 0.013	*0.077 ± 0.009

Note: SL – stride length; SF – stride frequency; T_c_ – contact time; T_f_ – flight time; K_leg_ – leg stiffness; K_vert_ – vertical stiffness; F_max_ – maximal force; ΔY_c_ – vertical oscillation during the contact; Δ*L* – vertical oscillation during the step.

## Discussion

The purpose of this study was to test the hypothesis that the running CoT would increase when preceded by cycling exercise associated with decreases in W_tot_, K_leg_, and K_vert_, resulting in reduction in *Eff*. The hypothesis was refuted because the *Eff* remains unchanged after cycling in middle-level triathletes, although there was increase in CoT, adding that this decreasing of metabolic economy was related to mechanical adjustments. That is, middle-level triathletes systematically reduce their stride length and increase their stride frequency when running after cycling, in comparison to running without previous cycling, preserving mechanical efficiency and work. Therefore, the findings of similar K_leg_, and K_vert_ are partly in line with a previous study^8^, which reported that elite triathletes preserved their neuromuscular control and running economy after cycling. Also, the maintenance of stiffness observed in our study, confirms previous rationale showing that a better stiffness regulation in the elite triathletes but not in their less successful counterparts^4^.

In these studies, however, the protocols vary greatly, and the reports are level-dependent. For example, in other studies comparing physiological parameters and performance after two previous cycling protocols in well-trained athletes, one protocol with constant intensity and another with variable intensity, the triathletes showed greater physiological deterioration in the race following the test with variable intensity of cycling^32^. The protocol of the present study kept the intensity of cycling constant with cycling power equivalent to 80% of the ventilatory threshold. It is important to consider the distance of the triathlon competition and the repercussions of these distances on adopted pacing strategies during specific disciplines within the triathlon^33^.

Our findings show that the inclusion of cycling prior to running preserved *Eff* at the end of the submaximal running test (Fig. 3). *Eff* is determined as the ratio between the W_tot_ and CoT, and the reduction in *Eff* in the control condition (without previous cycling) was mainly explained by the decrease in total mechanical work, consequently due to reduced W_ext_ during the final section of the test (Fig. 1). In a previous study, middle-level triathletes showed no difference in W_ext_ with and without previous cycling, but the running protocol was seven minutes long and the mechanical and metabolic parameters were collected only once during each running test^9^. The reasons for better metabolic economy in high level and lower metabolic economy in middle level triathletes found in previous studies^4^ are yet to be solved. Our study shows that, in middle level triathletes, the metabolic economy is deteriorated in association to lower stride length. Thus, our results are in line with previous studies analyzing short-duration tests but showed differences when evaluated over a longer time. In the present study, the running time was relatively long and the intensity was approximately 70% ventilatory threshold. The W_tot_ during the race with previous cycling is the main explanation for the better *Eff* in the final section because, in the present study, the CoT remained constant during all stages. Again, in the final section, the W_tot_ was higher during the race with previous cycling. Candau *et al*.^34^ found an increase in W_tot_ and O_2_ consumption from the beginning to the end of the running test at a high-intensity constant speed until exhaustion in triathletes. Avogadro *et al*. (2003), also using a constant velocity protocol until exhaustion in runners and triathletes, found an increase in O_2_ consumption at the end of the test, but with no change in mechanical cost. The slow component of $\dot{{\rm{V}}}$O_2_ in progressive race velocity was previously investigated and it was demonstrated that the slow component of $\dot{{\rm{V}}}$O_2_ was not a result of changes in the production of mechanical work^35^. However, in these studies, the metabolic intensity was higher and the exercise duration was shorter than in our study.

Stiffness at the end of the running test was lower with previous cycling in comparison to control conditions. This mechanical alteration may have helped to maintain the *Eff*, demonstrating a positive adaptation factor of triathletes to previous cycling. Borrani *et al*.^35^ suggested that the slow component of $\dot{{\rm{V}}}$O_2_ during running may be due to changes in the storage and reuse of elastic energy. Studies that evaluated races over a longer period, for example, 5 h^36^ and 24 h^37^ races, found an increase in K_leg_ and K_vert_ at the end of the races, and these changes may be interpreted as a smoother technique with a decrease in the vertical oscillation of the mass-spring system. In addition, the stiffness of the mass-spring system has a negative correlation with running CoT and the athlete was more economical due to running mechanics^38^.

Training specificity is an important factor to determine athlete performance. Triathletes who train sequentially in the three modalities have specific mechanical^39^ and metabolic^40^ adaptations in contrast to athletes who train, for example, running in isolation. The responses in the running CoT after pedaling are highly controversial. Some findings indicate lower values^9^ and others indicate similar values^8^ in high-level triathletes. Even higher values of CoT have been found in lower-level triathletes^18,41–46^. One mechanism for deterioration of metabolic economy is the depletion of muscle glycogen^46^.

Stride length and frequency presented an inverse relationship, with a short length and a high frequency when prerace cycling was performed. Although, changes in CoT have been observed between races with and without prior cycling, the *Eff* was preserved, demonstrating that athletes altered their mechanics to optimize their CoT, since runners naturally opt for a combination of stride frequency and stride length to minimize metabolic cost^47,48^. This mechanism has been recently shown by Lussiana *et al*.^49^ where the strategy of energy minimization is limiting the vertical oscillation of body center of mass to promote forward progression throughout a low duty factor (relatively long time contact and short flight time) resulting in a running technique less bouncy^49^. In addition, the increased stride frequency and reduced stride length seem to reduce the magnitude of the biomechanical factors associated with running injuries^50^ and, therefore, this mechanical alteration seems also to occur as a protective factor for triathletes. The F_max_, ΔL and Δyc during the previous cycling test presented lower values at the end compared to the isolated run, as found by Morin *et al*.^51^ after a long run (24 h), corroborating with the hypothesis of a change in mechanics for *Eff* maintenance and mechanical work after the moderate intensity cycling protocol. Another interpretation, from an integrative point-of-view, is that the maintenance of mechanical work and *Eff* accompanied by an increase in CoT provides a clear evidence that the muscle efficiency, in turn, constituted by the efficiencies of phosphorylative coupling and contraction coupling, is reduced after cycling^52^. Findings from experiments using just cycling showed a reduction on gross mechanical efficiency from time-trails at moderate intensities, confirming the deterioration on muscle efficiency as a candidate in our study^53^. The practical application to middle level athletes from our findings is that reductions on stride length accompanied by increases on stride rate in order to maintain the running speed after cycling bouts may be motor strategies to preserve the mechanical efficiency but increasing the metabolic cost of running. Future studies should be conducted to assess the effect of interventions, as strength training^54^ or post-activation potentiation^55^ on these alterations.

An important limitation of the study is that the constant velocity protocol adopted in the submaximal test has less ecological validity when compared to field tests. However, the laboratory tests are able to isolate factors that would alter the variables as a function of speed variation that modifies the biomechanical and physiological components. In a study comparing the behavior of the mass-spring system in four stages at ≈2,400 m − 4,800 m − 7,200 m- 9,600 m during a 10 km triathlon race, K_leg_ and K_vert_ variables decreased from the 1st to 3rd stages and increased in the 4th stage. This finding is related to the change in velocity that occurred during the test^17^. Keeping the speed constant, within a controlled intensity range, we were able to isolate this factor and observe that previous cycling also influences the stiffness of the mass-spring system.

## Conclusion

Prerace cycling increase the running CoT in middle-level triathletes. The mechanical work and stiffness of the spring-mass system are maintained throughout a constant speed test when cycling at moderate intensity, demonstrating that prerace cycling can contribute to the maintenance of mechanical efficiency in triathletes. In addition, the stride length decreases and the stride frequency increases with previous cycling, demonstrating an adaptation of the technique for *Eff* maintenance.

## Supplementary information

Supplementary Dataset

Supplementary Information

## References

[1] Cavagna GA, Kaneko M (1977). Mechanical work and efficiency in level walking and running. J. Physiol..

[2] Farris DJ, Sawicki GS (2012). The mechanics and energetics of human walking and running: a joint level perspective. J. R. Soc. Interface.

[3] Taboga Paolo, Lazzer Stefano, Fessehatsion Rezene, Agosti Fiorenza, Sartorio Alessandro, di Prampero Pietro E. (2012). Energetics and mechanics of running men: the influence of body mass. European Journal of Applied Physiology.

[4] Roberts TJ, Kram R, Weyand PG, Taylor CR (1998). Energetics of bipedal running. I. Metabolic cost of generating force. J. Exp. Biol..

[5] McBride JM (2015). Index of mechanical efficiency in competitive and recreational long distance runners. J. Sports Sci..

[6] Kyröläinen, H., Belli, A. & Komi, P. V. Biomechanical factors affecting running economy. *Med. Sci. Sports Exerc*. 1330–1337 10.1097/00005768-200108000-00014 (2001). 10.1097/00005768-200108000-00014 11474335

[7] Fletcher Jared R., Esau Shane P., MacIntosh Brian R. (2010). Changes in tendon stiffness and running economy in highly trained distance runners. European Journal of Applied Physiology.

[8] Bonacci J, Saunders PU, Alexander M, Blanch P, Vicenzino B (2011). Neuromuscular control and running economy is preserved in elite international triathletes after cycling. Sport. Biomech..

[9] Millet GP, Millet GY, Hofmann MD, Candau RB (2000). Alterations in running economy and mechanics after maximal cycling in triathletes: Influence of performance level. Int. J. Sports Med..

[10] Blickhan R (1989). The spring-mass model for running and hopping. J. Biomech..

[11] Fletcher JR, MacIntosh BR (2015). Achilles tendon strain energy in distance running: consider the muscle energy cost. J. Appl. Physiol..

[12] Ker RF, Bennett MB, Bibby SR, Kester RC, Alexander RM (1987). The spring in the arch of the human foot. Nature.

[13] Stearne SM (2016). The Foot’s Arch and the Energetics of Human Locomotion. Sci. Rep..

[14] da Rosa, R. G. *et al*. Landing-Takeoff Asymmetries Applied to Running Mechanics: A New Perspective for Performance. *Front. Physiol*. 10.3389/fphys.2019.00415 (2019). 10.3389/fphys.2019.00415 PMC6477028 31040793

[15] Farley CT, González O (1996). Leg stiffness and stride frequency in human running. J. Biomech..

[16] Markström JL, Olsson CJ (2013). Countermovement jump peak force relative to body weight and jump height as predictors for sprint running performances: (In)homogeneity of track and field athletes?. J. Strength Cond. Res..

[17] Le Meur Y (2013). Spring-mass behaviour during the run of an international triathlon competition. Int. J. Sports Med..

[18] Rabita G, Slawinski J, Girard O, Bignet F, Hausswirth C (2011). Spring-mass behavior during exhaustive run at constant velocity in elite triathletes. Med. Sci. Sports Exerc..

[19] Wahrlich Vivian, Anjos Luiz A., Going Scott B., Lohman Timothy G. (2006). Validation of the VO2000 calorimeter for measuring resting metabolic rate. Clinical Nutrition.

[20] Chou Li-Shan, Kaufman Kenton R., Brey Robert H., Draganich Louis F. (2001). Motion of the whole body's center of mass when stepping over obstacles of different heights. Gait & Posture.

[21] Nardello F, Ardigò LP, Minetti AE (2011). Measured and predicted mechanical internal work in human locomotion. Hum. Mov. Sci..

[22] HOWLEY EDWARD T., BASSETT DAVID R., WELCH HUGH G. (1995). Criteria for maximal oxygen uptake. Medicine & Science in Sports & Exercise.

[23] Beaver W. L., Wasserman K., Whipp B. J. (1986). A new method for detecting anaerobic threshold by gas exchange. Journal of Applied Physiology.

[24] Zatsiorsky, V. M. M. Kinematics of Human Motion. *American Journal of Human Biology* 99.1998/zatsiorsky.0880116765 (1998).

[25] Willems Pa, Cavagna GA, Heglund NC (1995). External, internal and total work in human locomotion. J. Exp. Biol..

[26] Nummela AT (2006). Neuromuscular factors determining 5 km running performance and running economy in well-trained athletes. Eur. J. Appl. Physiol..

[27] Tartaruga MP (2012). The relationship between running economy and biomechanical variables in distance runners. Res. Q. Exerc. Sport.

[28] Morin J-B, Dalleau G, Kyröläinen H, Jeannin T, Belli A (2005). A Simple Method for Measuring Stiffness during Running. J. Appl. Biomech..

[29] di Prampero PE, Atchou G, Brückner J-C, Moia C (1986). The energetics of endurance running. Eur. J. Appl. Physiol. Occup. Physiol..

[30] Saunders PU, Pyne DB, Telford RD, Hawley JA (2004). Factors affecting running economy in trained distance runners. Sport. Med..

[31] Péronnet F, Massicotte D (1991). Table of nonprotein respiratory quotient: an update. Can. J. Sport Sci..

[32] Etxebarria N, Anson JM, Pyne DB, Ferguson RA (2014). High-intensity cycle interval training improves cycling and running performance in triathletes. Eur. J. Sport Sci..

[33] Wu SSX (2015). Pacing strategies during the swim, cycle and run disciplines of sprint, Olympic and half-Ironman triathlons. Eur. J. Appl. Physiol..

[34] Candau R (1998). Energy cost and running mechanics during a treadmill run to voluntary exhaustion in humans. Eur. J. Appl. Physiol. Occup. Physiol..

[35] Borrani F (2003). Does the Mechanical Work in Running Change during the VO2 slow component?. Med. Sci. Sports Exerc..

[36] Degache F (2013). Changes in running mechanics and spring-mass behaviour induced by a 5-hour hilly running bout. J. Sports Sci..

[37] Morin JB, Tomazin K, Edouard P, Millet GY (2011). Changes in running mechanics and spring-mass behavior induced by a mountain ultra-marathon race. J. Biomech..

[38] Dalleau, G., Belli, A, Bourdin, M. & Lacour, J. R. The spring-mass model and the energy cost of treadmill running. *Eur. J. Appl. Physiol. Occup. Physiol*. 10.1007/s004210050330 (1998). 10.1007/s004210050330 9535587

[39] Luna NMS (2012). Isokinetic analysis of ankle and ground reaction forces in runners and triathletes. Clinics (Sao Paulo)..

[40] Bentley DJ, Millet GP, Vleck VE, McNaughton LR (2002). Specific Aspects of Contemporary Triathlon. Sport. Med..

[41] kreider RB, Boone T, Thompson WR, Burkes S, Cortes CW (1988). Cardiovascular and thermal responses of triathlon performance. Med. Sci. Sport. Exerc..

[42] Guezennec CY, Vallier JM, Bigard AX, Durey A (1996). Increase in energy cost of running at the end of a triathlon. Eur. J. Appl. Physiol. Occup. Physiol..

[43] Hausswirth C, Bigard AX, Berthelot M, Thomaidis M, Guezennec CY (1996). Variability in energy cost of running at the end of a triathlon and a marathon. Int. J. Sports Med..

[44] Hausswirth C, Bigard AX, Guezennec CY (1997). Relationships between running mechanics and energy cost of running at the end of a triathlon and a marathon. Int. J. Sports Med..

[45] Hue O, Le Gallais D, Chollet D, Boussana A, Prefaut C (1998). The influence of prior cycling on biomechanical and cardiorespiratory response profiles during running in triathletes. Eur. J. Appl. Physiol. Occup. Physiol..

[46] Suriano R, Bishop D (2010). Combined cycle and run performance is maximised when the cycle is completed at the highest sustainable intensity. Eur. J. Appl. Physiol..

[47] Cavanagh PR, Williams KR (1982). The effect of stride length variation on oxygen uptake during distance running. Med. Sci. Sports Exerc..

[48] Hunter I, Smith GA (2007). Preferred and optimal stride frequency, stiffness and economy: Changes with fatigue during a 1-h high-intensity run. Eur. J. Appl. Physiol..

[49] Lussiana, T., Patoz, A., Gindre, C., Mourot, L. & Hebert-Losier, K. The implications of time on the ground on running economy: less is not always better. *J. Exp. Biol*. **222**, (2019). 10.1242/jeb.192047 30787136

[50] Schubert AG, Kempf J, Heiderscheit BC (2014). Influence of Stride Frequency and Length on Running Mechanics: A Systematic Review. Sports Health.

[51] Morin JB, Samozino P, Edouard P, Tomazin K (2011). Effect of fatigue on force production and force application technique during repeated sprints. J. Biomech..

[52] Peyré-Tartaruga, L. A. & Coertjens, M. Locomotion as a powerful model to study integrative physiology: Efficiency, economy, and power relationship. *Front. Physiol*. 10.3389/fphys.2018.01789 (2018). 10.3389/fphys.2018.01789 PMC6297284 30618802

[53] Passfield L, Doust JH (2000). Changes in cycling efficiency and performance after endurance exercise. Med. Sci. Sports Exerc..

[54] Millet GP, Jaouen B, Borrani F, Candau R (2002). Effects of concurrent endurance and strength training on running economy and.VO2 kinetics. Med. Sci. Sports Exerc..

[55] Boullosa D, Del Rosso S, Behm DG, Foster C (2018). Post-activation potentiation (PAP) in endurance sports: A review. Eur. J. Sport Sci..

