# Supplementary material for: Running Stride Length And Rate Are Changed And Mechanical Efficiency Is Preserved After Cycling In Middle-Level Triathletes
Source: Sci Rep. 2019 Dec 5;9:18422. doi: 10.1038/s41598-019-54912-6 (PMC6895242; doi:10.1038/s41598-019-54912-6)
Supplement: Supplementary file 1 — Supplementary Dataset [file 41598_2019_54912_MOESM1_ESM.docx]

Dataset of the study:

# RUNNING STRIDE LENGTH AND RATE ARE CHANGED BUT MECHANICAL EFFICIENCY IS PRESERVED AFTER CYCLING IN MIDDLE-LEVEL TRIATHLETES

Table 1 – Mechanical efficiency (Eff)

| **mechanical efficiency** | Eff |  |  |  |  |  |  |  |
| --- | --- | --- | --- | --- | --- | --- | --- | --- |
| **subjects** | **1c bike** | **2c** | **3c** | **4c** | **1s/bike** | **2s** | **3s** | **4s** |
| 1 | 0.61 | 0.58 | 0.53 | 0.52 | 0.51 | 0.45 | 0.46 | 0.42 |
| 2 | 0.49 | 0.48 | 0.47 | 0.50 | 0.56 | 0.53 | 0.51 | 0.58 |
| 3 | 0.70 | 0.73 | 0.68 | 0.65 | 0.50 | 0.53 | 0.55 | 0.70 |
| 4 | 0.51 | 0.47 | 0.49 | 0.49 | 0.55 | 0.61 | 0.54 | 0.52 |
| 5 | 0.55 | 0.53 | 0.49 | 0.47 | 0.79 | 0.71 | 0.72 | 0.60 |
| 6 | 0.66 | 0.71 | 0.69 | 0.66 | 0.62 | 0.70 | 0.70 | 0.63 |
| 7 | 0.66 | 0.65 | 0.66 | 0.58 | 0.54 | 0.59 | 0.66 | 0.56 |
| 8 | 0.63 | 0.62 | 0.76 | 0.60 | 0.65 | 0.66 | 0.74 | 0.59 |
| 9 | 0.64 | 0.59 | 0.61 | 0.59 | 0.65 | 0.91 | 0.88 | 0.68 |
| 10 | 0.62 | 0.66 | 0.56 | 0.59 | 0.64 | 0.68 | 0.64 | 0.63 |
| 11 | 0.62 | 0.63 | 0.65 | 0.65 | 0.71 | 0.65 | 0.73 | 0.65 |
| 12 | 0.52 | 0.54 | 0.50 | 0.45 | 0.83 | 0.73 | 0.79 | 0.73 |
| 13 | 0.84 | 0.69 | 0.70 | 0.58 | 0.85 | 0.67 | 0.81 | 0.60 |
| mean | 0.62 | 0.61 | 0.60 | 0.56 | 0.65 | 0.65 | 0.67 | 0.61 |
| sd | 0.09 | 0.08 | 0.10 | 0.07 | 0.12 | 0.11 | 0.13 | 0.08 |

Table 2 – Cost-of-Transport (CoT) in J kg^-1^ m^-1^.

| **cost of transport** | CoT |  |  |  |  |  |  |  |
| --- | --- | --- | --- | --- | --- | --- | --- | --- |
| **subjects** | **1c bike** | **2c** | **3c** | **4c** | **1s/bike** | **2s** | **3s** | **4s** |
| 1 | 3.88 | 4.24 | 4.37 | 4.26 | 4.15 | 4.19 | 4.02 | 4.02 |
| 2 | 4.27 | 4.12 | 4.27 | 4.12 | 4.11 | 4.21 | 4.15 | 4.03 |
| 3 | 3.21 | 3.28 | 3.28 | 3.28 | 3.56 | 3.62 | 3.45 | 2.67 |
| 4 | 4.56 | 4.84 | 4.50 | 4.37 | 3.48 | 3.54 | 3.71 | 3.73 |
| 5 | 3.90 | 4.23 | 4.55 | 4.56 | 2.97 | 3.23 | 3.22 | 3.35 |
| 6 | 3.42 | 3.16 | 3.38 | 3.39 | 3.40 | 3.53 | 3.33 | 3.34 |
| 7 | 3.64 | 3.73 | 3.70 | 3.74 | 3.62 | 3.65 | 3.48 | 3.42 |
| 8 | 3.21 | 3.30 | 3.13 | 3.22 | 3.44 | 3.58 | 3.22 | 3.48 |
| 9 | 3.85 | 4.09 | 4.00 | 4.05 | 2.24 | 2.67 | 2.75 | 2.85 |
| 10 | 3.45 | 3.72 | 3.70 | 3.52 | 2.78 | 3.11 | 3.22 | 2.96 |
| 11 | 3.23 | 3.17 | 3.07 | 2.98 | 3.02 | 2.43 | 2.77 | 2.76 |
| 12 | 3.63 | 3.76 | 3.80 | 4.03 | 2.98 | 3.13 | 3.01 | 2.96 |
| 13 | 2.66 | 2.97 | 2.92 | 2.96 | 2.80 | 2.88 | 3.22 | 2.81 |
|  | 3.61 | 3.74 | 3.74 | 3.73 | 3.27 | 3.37 | 3.35 | 3.26 |
|  | 0.50 | 0.55 | 0.56 | 0.54 | 0.54 | 0.53 | 0.42 | 0.47 |

Table 3 – Internal mechanical work (Wint) in J kg^-1^ m^-1^.

| **internal mechanical work** | Wint |  |  |  |  |  |  |  |
| --- | --- | --- | --- | --- | --- | --- | --- | --- |
| **Subjects** | **1c bike** | **2c** | **3c** | **4c** | **1s/bike** | **2s** | **3s** | **4s** |
| 1 | 0.60 | 0.56 | 0.57 | 0.59 | 0.54 | 0.52 | 0.56 | 0.48 |
| 2 | 0.56 | 0.55 | 0.59 | 0.67 | 0.52 | 0.54 | 0.58 | 0.53 |
| 3 | 0.58 | 0.55 | 0.60 | 0.62 | 0.61 | 0.62 | 0.57 | 0.55 |
| 4 | 0.60 | 0.55 | 0.61 | 0.54 | 0.62 | 0.62 | 0.54 | 0.52 |
| 5 | 0.65 | 0.65 | 0.61 | 0.61 | 0.73 | 0.64 | 0.64 | 0.61 |
| 6 | 0.55 | 0.55 | 0.55 | 0.54 | 0.52 | 0.53 | 0.54 | 0.51 |
| 7 | 0.58 | 0.57 | 0.55 | 0.63 | 0.56 | 0.59 | 0.56 | 0.55 |
| 8 | 0.64 | 0.60 | 0.58 | 0.63 | 0.58 | 0.55 | 0.55 | 0.60 |
| 9 | 0.54 | 0.53 | 0.53 | 0.53 | 0.55 | 0.56 | 0.53 | 0.53 |
| 10 | 0.58 | 0.63 | 0.59 | 0.61 | 0.58 | 0.60 | 0.58 | 0.57 |
| 11 | 0.62 | 0.64 | 0.61 | 0.67 | 0.63 | 0.58 | 0.54 | 0.58 |
| 12 | 0.65 | 0.64 | 0.65 | 0.63 | 0.62 | 0.57 | 0.59 | 0.62 |
| 13 | 0.53 | 0.53 | 0.54 | 0.57 | 0.48 | 0.51 | 0.51 | 0.55 |
| mean | 0.59 | 0.58 | 0.58 | 0.60 | 0.58 | 0.57 | 0.56 | 0.55 |
| sd | 0.04 | 0.05 | 0.03 | 0.05 | 0.06 | 0.04 | 0.03 | 0.04 |

Table 4 – External mechanical work (Wext) in J kg^-1^ m^-1^.

| **external mechanical work** | Wext |  |  |  |  |  |  |  |
| --- | --- | --- | --- | --- | --- | --- | --- | --- |
| **subjects** | **1c bike** | **2c** | **3c** | **4c** | **1s/bike** | **2s** | **3s** | **4s** |
| 1 | 1.76 | 1.9 | 1.76 | 1.62 | 1.58 | 1.36 | 1.3 | 1.21 |
| 2 | 1.53 | 1.42 | 1.43 | 1.39 | 1.78 | 1.70 | 1.55 | 1.80 |
| 3 | 1.66 | 1.84 | 1.63 | 1.51 | 1.17 | 1.32 | 1.33 | 1.31 |
| 4 | 1.71 | 1.74 | 1.61 | 1.59 | 1.29 | 1.55 | 1.48 | 1.41 |
| 5 | 1.51 | 1.58 | 1.63 | 1.52 | 1.61 | 1.65 | 1.67 | 1.41 |
| 6 | 1.69 | 1.71 | 1.77 | 1.7 | 1.58 | 1.95 | 1.79 | 1.58 |
| 7 | 1.81 | 1.87 | 1.87 | 1.55 | 1.41 | 1.57 | 1.75 | 1.37 |
| 8 | 1.38 | 1.44 | 1.79 | 1.30 | 1.66 | 1.80 | 1.83 | 1.45 |
| 9 | 1.91 | 1.88 | 1.92 | 1.87 | 1.89 | 1.87 | 1.9 | 1.41 |
| 10 | 1.55 | 1.84 | 1.48 | 1.46 | 1.21 | 1.51 | 1.49 | 1.29 |
| 11 | 1.38 | 1.35 | 1.39 | 1.28 | 1.53 | 1.85 | 1.5 | 1.21 |
| 12 | 1.24 | 1.38 | 1.26 | 1.20 | 1.84 | 1.72 | 1.79 | 1.54 |
| 13 | 1.72 | 1.51 | 1.51 | 1.15 | 1.9 | 1.43 | 2.1 | 1.14 |
| mean | 1.60 | 1.65 | 1.62 | 1.47 | 1.57 | 1.64 | 1.65 | 1.39 |
| sd | 0.19 | 0.21 | 0.20 | 0.21 | 0.25 | 0.20 | 0.23 | 0.18 |

Table 5 – Total mechanical work (Wtot) in J kg^-1^ m^-1^.

| **total mechanical work** | Wtot |  |  |  |  |  |  |  |
| --- | --- | --- | --- | --- | --- | --- | --- | --- |
| **Subjects** | **1c bike** | **2c** | **3c** | **4c** | **1s/bike** | **2s** | **3s** | **4s** |
| 1 | 2.36 | 2.46 | 2.33 | 2.21 | 2.12 | 1.88 | 1.86 | 1.69 |
| 2 | 2.09 | 1.97 | 2.02 | 2.06 | 2.30 | 2.24 | 2.13 | 2.33 |
| 3 | 2.24 | 2.39 | 2.23 | 2.13 | 1.78 | 1.94 | 1.90 | 1.86 |
| 4 | 2.31 | 2.29 | 2.22 | 2.13 | 1.91 | 2.17 | 2.02 | 1.93 |
| 5 | 2.16 | 2.23 | 2.24 | 2.13 | 2.34 | 2.29 | 2.31 | 2.02 |
| 6 | 2.24 | 2.26 | 2.32 | 2.24 | 2.10 | 2.48 | 2.33 | 2.09 |
| 7 | 2.39 | 2.44 | 2.42 | 2.18 | 1.97 | 2.16 | 2.31 | 1.92 |
| 8 | 2.02 | 2.04 | 2.37 | 1.93 | 2.24 | 2.35 | 2.38 | 2.05 |
| 9 | 2.45 | 2.41 | 2.45 | 2.40 | 2.44 | 2.43 | 2.43 | 1.94 |
| 10 | 2.13 | 2.47 | 2.07 | 2.07 | 1.79 | 2.11 | 2.07 | 1.86 |
| 11 | 2.00 | 1.99 | 2.00 | 1.95 | 2.16 | 2.43 | 2.04 | 1.79 |
| 12 | 1.89 | 2.02 | 1.91 | 1.83 | 2.46 | 2.29 | 2.38 | 2.16 |
| 13 | 2.25 | 2.04 | 2.05 | 1.72 | 2.38 | 1.94 | 2.61 | 1.69 |
| mean | 2.19 | 2.23 | 2.20 | 2.07 | 2.15 | 2.21 | 2.21 | 1.95 |
| sd | 0.17 | 0.20 | 0.17 | 0.18 | 0.23 | 0.20 | 0.23 | 0.18 |

Abbreviations

| Eff | mechanical efficiency |
| --- | --- |
| 1c bike | first path (3-5th minute) after cycling condition |
| 2c | second path (8-10th minute) after cycling condition |
| 3c | third path (13-15th minute) after cycling condition |
| 4c | fourth path path (18-20th minute) after cycling condition |
| 1s/bike | first path (3-5th minute) control condition |
| 2s | second path (8-10th minute) control condition |
| 3s | third path (13-15th minute) control condition |
| 4s | fourth path path (18-20th minute) control condition |
| Wint | internal mechanical work |
| Wext | external mechanical work |
| CoT | cost of transport |
| Wtot | total mechanical work |
