# Supplementary material for: Running Stride Length And Rate Are Changed And Mechanical Efficiency Is Preserved After Cycling In Middle-Level Triathletes
Source: Sci Rep. 2019 Dec 5;9:18422. doi: 10.1038/s41598-019-54912-6 (PMC6895242; doi:10.1038/s41598-019-54912-6)
Supplement: Supplementary file 2 — Supplementary Information [file 41598_2019_54912_MOESM2_ESM.docx]

Electronic supplementary material 02 from da Rosa et al.

# RUNNING STRIDE LENGTH AND RATE ARE CHANGED AND MECHANICAL EFFICIENCY IS PRESERVED AFTER CYCLING IN MIDDLE-LEVEL TRIATHLETES

No differences in the metabolic power (P_met_) were found between the conditions with and without previous cycling and among the test stages. The mechanical power (P_mec_) was lower at the final stage compared to the second and third stages, with no differences between running with and without previous cycling

.

**ESM 2.** Metabolic and mechanical power data (Mean ± standard-deviation) during the 20-minute run at 14 km.h^-1^.

|  | Pmet (W.kg^-1^) | | Pmech (W.kg^-1^) | |
| --- | --- | --- | --- | --- |
| Time (min) | Control | Post-Cycling | Control | Post-Cycling |
| 3’-5’ | 13.29 ± 1.76^a^ | 13.78 ± 2.17^a^ | 8.26 ± 0.60^a^ | 8.11 ± 0.85^a,b,c^ |
| 8’-10’ | 13.53 ± 2.07^a^ | 14.34 ± 2.16^a^ | 8.42 ± 0.71^a^ | 8.41 ± 0.79^a,b^ |
| 13’-14’ | 13.46 ± 1.81^a^ | 14.28 ± 2.73^a^ | 8.31 ± 0.63^a^ | 8.40 ± 0.85^a,b^ |
| 18’-20’ | 13.23 ± 1.93^a^ | 14.34 ± 2.16^a^ | 7.82 ± 0.66^a^ | 7.31 ± 0.67^c^ |

Note – Pmet: metabolic power; Pmech: mechanical power; Control: without preceding-cycling condition; Post-Cycling: with preceding-cycling condition. Different letters indicate significant differences between paths of submaximal running test (p < 0.05).
